# Die HNO-fachärztliche Weiterbildung in Deutschland im Kontext der Krankenhausreform

**DOI:** 10.1007/s00106-026-01737-1

**Published:** 2026-02-03

**Authors:** Thomas K. Hoffmann, Janina Hahn, Thomas Deitmer, Martin Jäckel, Marcus Neudert, Timo Stöver

**Affiliations:** 1 Klinik für Hals‑, Nasen‑, Ohrenheilkunde, Kopf- und Halschirurgie, Universitätsklinikum Ulm, Frauensteige 12, 89075 Ulm, Deutschland; 2 Deutsche Gesellschaft für Hals-Nasen-Ohren-Heilkunde, Kopf- und Hals-Chirurgie e. V., Bonn, Deutschland; 3 Klinik für Hals‑, Nasen‑, Ohrenheilkunde, Helios Kliniken Schwerin, Schwerin, Deutschland; 4 Klinik für Hals‑, Nasen‑, Ohrenheilkunde, Carl Gustav Carus Universität Dresden, Dresden, Deutschland; 5 Klinik für Hals‑, Nasen‑, Ohrenheilkunde, Universitätsmedizin Frankfurt a. M., Goethe-Universität Frankfurt a. M., Frankfurt a.M., Deutschland

**Keywords:** Fachärztliche Weiterbildung, HNO-Heilkunde, Klinikreform, Stationäre Versorgung, Ambulante Versorgung, Resident training, Otorhinolaryngology, Hospital reform, Inpatient care, Ambulatory care

## Abstract

**Hintergrund:**

In der Hals‑, Nasen‑, Ohrenheilkunde (HNO) erfolgt die Facharztweiterbildung überwiegend in stationären Einrichtungen. Vor dem Hintergrund der aktuellen Strukturreformen im Krankenhaussektor (Krankenhausversorgungsverbesserungsgesetz/KHVVG) ist es notwendig, grundlegende Strukturdaten dieser Weiterbildungsstätten zu erfassen. Gegenwärtig existiert keine zentrale Erfassung der Anzahl der stationären Weiterbildungsstätten, der Weiterzubildenden oder Weiterbildungsberechtigten und der Weiterbildungszeiten in Deutschland. Ziel dieser Arbeit war es, eine Standortbestimmung für die genannten Variablen zu erstellen, um eine Grundlage für die Bewertung der möglichen Auswirkungen der Krankenhausreform zu schaffen.

**Material und Methoden:**

Die Deutsche Gesellschaft für Hals-Nasen-Ohren-Heilkunde, Kopf- und Hals-Chirurgie (DGHNO-KHC) führte eine deutschlandweite digitale Umfrage unter den Leitungen stationärer HNO-Hauptabteilungen durch. Erfasst wurden strukturelle und kapazitätsbezogene Merkmale im Kontext der fachärztlichen Weiterbildung.

**Ergebnisse:**

Es wurden 95 vollständig beantwortete Fragebögen ausgewertet (Rücklaufquote 54,9 %). Alle Bundesländer waren vertreten (32 universitäre und 63 nichtuniversitäre Abteilungen). Im Erhebungsjahr 2024 berichteten 84 Kliniken (88,4 %) von mehr als 1500 stationären Fällen. Pro Einrichtung verfügten im Durchschnitt 1,5 Fachärzte über eine Weiterbildungsermächtigung. Die Zahl der Weiterbildungsassistenten blieb in 56,8 % der Kliniken stabil und nahm in 32,6 % zu. Insgesamt hatten 96,8 % der Einrichtungen die volle Weiterbildungsermächtigung über 5 Jahre.

**Schlussfolgerung:**

Die fachärztliche Weiterbildung in der Hals‑, Nasen‑, Ohrenheilkunde wird bundesweit breit und qualitativ hochwertig durch stationäre Einrichtungen getragen. Nach den vorliegenden Daten ist auch unter Berücksichtigung möglicher Auswirkungen des KHVVG von einer weiterhin stabilen und leistungsfähigen Weiterbildungsstruktur auszugehen, die wesentlich zur Sicherstellung der fachärztlichen Versorgung in Deutschland beiträgt.

## Abstract (en)

**Background:**

In otorhinolaryngology, the majority of specialist training takes place in inpatient facilities. Against the backdrop of current structural reforms in the hospital sector, it is necessary to collect fundamental structural data of these training institutions. To date, no centrally collected data exist that capture the number of inpatient training centers, the number of trainees and authorized trainers, or the scope of training accreditation. The aim of this study was therefore to establish a nationwide baseline assessment to support evaluation of the potential impact of the ongoing hospital reform.

**Materials and methods:**

The German Society of Oto-Rhino-Laryngology, Head and Neck Surgery (DGHNO-KHC) conducted a nationwide digital survey among the heads of ENT departments in Germany. The survey collected structural and capacity-related parameters relevant to specialist training.

**Results:**

A total of 95 fully completed questionnaires were returned (response rate 54.9%). All federal states were represented (32 university and 63 non-university hospitals). In 2024, 84 clinics (88,4 %) reported more than 1500 inpatient cases. On average, 1.5 physicians per institution held formal authorization to conduct specialist training. The number of trainees remained stable in 56.8% of clinics and increased in 32.6%. Overall, 96.8% of departments had full accreditation for the complete 5‑year training period.

**Conclusion:**

Otorhinolaryngology specialist training in Germany is broadly available and delivered at a consistently high level within hospitals. Based on the collected data, a stable and robust training structure can be expected to persist, even when considering potential effects of the Hospital Care Improvement Act (*Krankenhausversorgungsverbesserungsgesetz*, KHVVG). These training capacities remain essential for ensuring nationwide high-quality specialist care.

Die ärztliche Weiterbildung ist eine der tragenden Säulen der medizinischen Versorgungsqualität in Deutschland. In der Hals‑, Nasen‑, Ohrenheilkunde (HNO) sichert sie nicht nur eine flächendeckende, qualitativ hochwertige Patientenversorgung, sondern auch die langfristige Nachwuchsgewinnung in einem zunehmend spezialisierungsintensiven Fachgebiet. Angesichts des demografischen Wandels, des Fachkräftemangels und struktureller Veränderungen in der stationären Versorgung steht die Weiterbildung vor erheblichen Herausforderungen.

Mit dem zum 1. Januar 2025 in Kraft getretenen Krankenhausversorgungsverbesserungsgesetz (KHVVG) wurde eine der umfassendsten Reformen der deutschen Krankenhauslandschaft der letzten Jahrzehnte umgesetzt [1]. Ziel des Gesetzes ist es, die Versorgungsqualität durch Einführung von Leistungsgruppen, verbindliche Strukturvorgaben und Mindestvorhaltezahlen zu sichern sowie eine stärker qualitätsorientierte und weniger fallzahlabhängige Finanzierung der stationären Leistungen zu etablieren. Für das Fachgebiet der HNO-Heilkunde definiert das KHVVG derzeit zwei Leistungsgruppen („Allgemeine HNO“ und „Cochlea-Implantate“) mit spezifischen Anforderungen an Personal- und Strukturqualität, darunter die Vorhaltung von mindestens drei Fachärzten in Vollzeitäquivalenten einschließlich 24/7-Rufbereitschaft.

Da die Facharztweiterbildung in der HNO u. a. aufgrund der operativen Anforderungen des Facharztkatalogs in relevanten Anteilen an stationären Einrichtungen erfolgt, sind durch die strukturelle Neuausrichtung der Krankenhauslandschaft auch erhebliche Auswirkungen auf die Weiterbildung zu erwarten. Zahlreiche kleinere Abteilungen und belegärztliche Strukturen, die bislang einen Teil der Facharztweiterbildung übernehmen, dürften die neuen personellen und organisatorischen Anforderungen nicht erfüllen können. Simulationen deuten darauf hin, dass bis zu 70 % der bestehenden HNO-(Beleg‑)Abteilungen unter den künftigen Voraussetzungen keinen Versorgungsauftrag mehr erhalten könnten [2]. Dies würde theoretisch zu einer deutlichen Reduktion der Weiterbildungsstätten und in der Folge zu einer Zentralisierung der Weiterbildung auf größere und universitäre Einrichtungen führen. Veränderungen in der Zahl und Größe der Kliniken, der personellen Ausstattung sowie in der Spezialisierung der Leistungsgruppen könnten damit unmittelbar die Ausbildungskapazitäten und den Umfang der Weiterbildungsermächtigungen beeinflussen.

Um die zu erwartenden Auswirkungen fundiert einschätzen zu können, sind zunächst objektive Daten zur aktuellen Weiterbildungssituation erforderlich. Hierzu gehören nicht nur die Anzahl der Weiterbildungsplätze im stationären und ambulanten Bereich, sondern auch die Berücksichtigung der Anforderungen des Facharztkatalogs HNO gemäß (Muster‑)Weiterbildungsordnung (MWBO). Ziel dieser Arbeit war es daher, eine bundesweite Standortbestimmung der aktuellen Weiterbildungssituation in der HNO-Heilkunde vorzunehmen. Die Erhebung und Analyse dieser Basisdaten soll eine Grundlage schaffen, um die potenziellen Auswirkungen der Krankenhausreform und insbesondere des KHVVG auf die zukünftige Weiterbildung im Fachgebiet objektiv bewerten zu können.

## Methodik

Grundlage der vorliegenden Arbeit war eine bundesweite Erhebung unter HNO-Kliniken, die als stationäre Weiterbildungsstätten im Jahr 2024 tätig waren. Erfasst wurden unter anderem der Umfang der Weiterbildungsermächtigung, das Bundesland der Einrichtung, die durchschnittliche Anzahl der Weiterbildungsassistenten, die Zahl der Weiterbildungsberechtigten sowie die Anzahl der durchgeführten Operationen und der ambulanten und stationären Behandlungsfälle.

Der Fragebogen umfasste Multiple-Choice-Formate, geschlossene Ja/Nein-Fragen sowie Fragen zur Angabe numerischer Werte. Bei jeder Frage bestand die Möglichkeit, „keine Angabe“ auszuwählen oder die Frage zu überspringen. In der Auswertung wurden beide Optionen gemeinsam als „keine Angabe“ berücksichtigt. Die angegebenen Prozentwerte beziehen sich zur besseren Vergleichbarkeit stets auf die Gesamtzahl der eingegangenen Fragebögen; zusätzlich wird bei jeder Frage ausgewiesen, wie viele Teilnehmende keine Antwort gegeben haben.

Der Fragebogen wurde mit SurveyMonkey (SurveyMonkey Europe UC, Irland) erstellt und im Juli 2025 durch die DGHNO-KHC digital versendet. Die Auswertung erfolgte anonymisiert und überwiegend deskriptiv mithilfe von Microsoft Excel.

## Ergebnisse

Die Rücklaufquote lag mit 95 beantworteten Fragebögen bei 54,9 %.

### Weiterbildungseinrichtungen

**Abb. 1:**
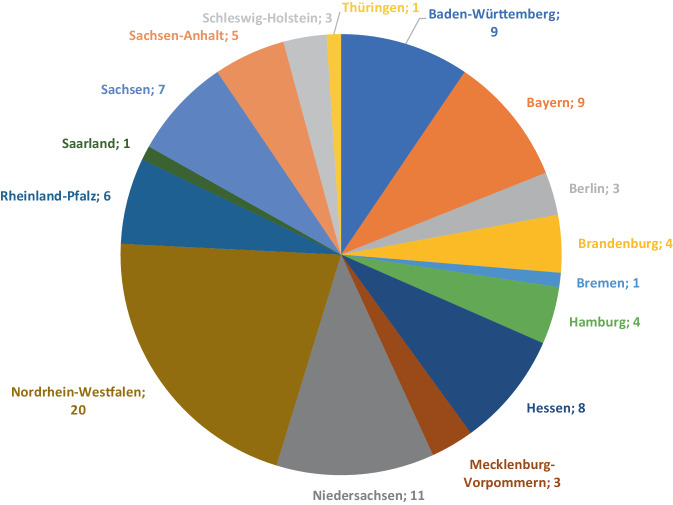
Bundesländer der an der Umfrage teilnehmenden HNO-Weiterbildungsstätten (*n* = 95)

Von den insgesamt 95 ausgewerteten Einrichtungen waren *n* = 32 (Rückmeldequote 80 %) HNO-Abteilungen an Universitätskliniken und *n* = 63 (Rückmeldequote 47,4 %) nichtuniversitäre HNO-Hauptabteilungen. Die regionale Verteilung der teilnehmenden Kliniken ist in Abb. 1 dargestellt.

Die Zahl der Weiterbildungsberechtigten (als Vollkraftäquivalente) lag im Jahr 2024 durchschnittlich bei 1,5 pro Einrichtung. Gleichzeitig waren an den befragten Abteilungen im Mittel 8,7 HNO-Fachärzte (Vollkraftäquivalente) beschäftigt.

Die durchschnittliche Zahl der Weiterbildungsassistenten betrug 9,4 Vollkraftäquivalente pro Klinik (Spanne: 3–28; *n* = 2 „keine Angabe“). In 31 Einrichtungen (32,6 %) nahm die Zahl der Weiterbildungsassistenten in den vergangenen Jahren zu, in 8 Einrichtungen (8,4 %) ab; 54 Kliniken (56,8 %) berichteten von stabilen Zahlen (*n* = 2 „keine Angabe“).

In 92 Abteilungen (96,8 %) lag die vollständige Weiterbildungsermächtigung für den Facharzt für HNO-Heilkunde über 5 Jahre vor; in einem Fall bestand eine dreijährige Ermächtigung (*n* = 2 „keine Angabe“).

Insgesamt befanden sich in den antwortenden Kliniken 818 Vollkraftäquivalente in HNO-Weiterbildung, was einem Durchschnitt von 9,4 pro Einrichtung entspricht.

### Behandlungs- und Weiterbildungsspektrum

Bezüglich der stationären Fallzahlen gaben 84 Einrichtungen (88,4 %) an, im Jahr 2024 mehr als 1500 stationäre Fälle (konservativ oder operativ) behandelt zu haben; 8 Kliniken (8,7 %) berichteten von weniger als 1500 Fällen (*n* = 3 „keine Angabe“).

**Abb. 2:**
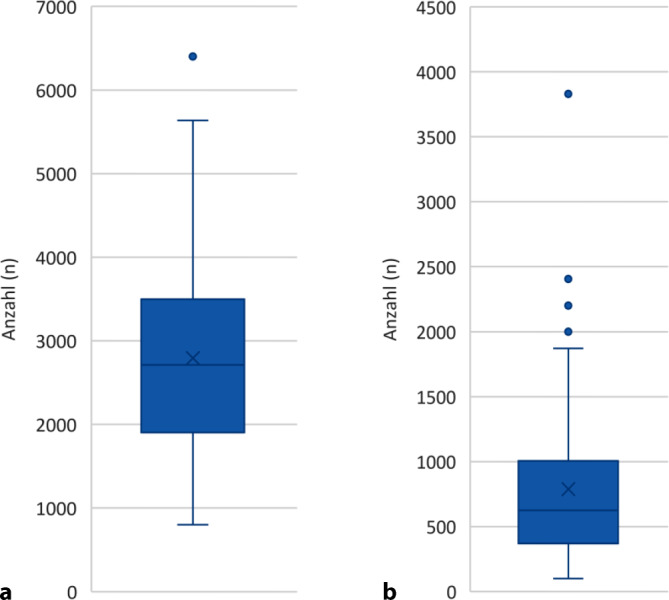
**a,** **b** Durchschnittliche Anzahl stationärer (**a**) und ambulanter (**b**) Operationen in den Weiterbildungsstätten im Jahr 2024

Die durchschnittliche Anzahl stationärer Operationen lag bei 2794 pro Einrichtung (*n* = 3 „keine Angabe“). Im ambulanten Bereich wurden durchschnittlich 787 Operationen durchgeführt (*n* = 3 „keine Angabe“; Abb. 2a, b).

Die teilnehmenden Einrichtungen behandelten darüber hinaus im Durchschnitt 10.590 ambulante Fälle (Spanne: 250–30.000; *n* = 4 „keine Angabe“).

## Diskussion

Ziel der vorliegenden Arbeit war es, aktuelle Daten zur Weiterbildungssituation an den stationären HNO-Einrichtungen in Deutschland zu erheben und auszuwerten. Vergleichbare systematische Erhebungen liegen unserer Kenntnis nach bislang nicht vor.

Die flächendeckende Verteilung der teilnehmenden Einrichtungen über alle Bundesländer sowie die hohen Fallzahlen belegen, dass die Weiterbildung im stationären Sektor weiterhin breit verankert ist. Im Erhebungsjahr 2024 behandelten 88,4 % der Kliniken mehr als 1500 stationäre Fälle; im Mittel wurden zudem 10.590 ambulante Fälle, 2794 stationäre und 787 ambulante Operationen durchgeführt. Nach aktuellen Simulationen ist davon auszugehen, dass diese Einrichtungen auch unter den Rahmenbedingungen des KHVVG bzw. des Krankenhausreformanpassungsgesetzes (KHAG) weiterhin eine maßgebliche Rolle sowohl in der Versorgung als auch in der Weiterbildung spielen werden [2].

In der Vergangenheit haben HNO-Hauptabteilungen kontinuierlich eine stabile Zahl von HNO-Fachärzten ausgebildet. Laut Ärztestatistik der Bundesärztekammer waren im Jahr 2024 insgesamt 6661 Fachärzte für HNO-Heilkunde beruflich tätig, davon 2721 Frauen. Die Zahl der jährlich neu anerkannten Fachärzte blieb in den letzten Jahren konstant (236 im Jahr 2022, 242 im Jahr 2023 und 239 im Jahr 2024) [3].

Die Umfrageergebnisse verdeutlichen zudem, dass in den teilnehmenden Kliniken insgesamt 818 Weiterbildungsassistenten (entspricht 9,4 Vollkraftäquivalenten pro Einrichtung) tätig waren und damit ein relevanter Anteil des bundesweiten Weiterbildungskontingents abgedeckt wird. Die Relation von Weiterbildungsassistenten zu Fachärzten liegt nahezu bei 1:1, was eine enge fachliche Betreuung ermöglicht. In 56,8 % der Kliniken blieb die Zahl der Weiterzubildenden stabil, in weiteren 32,6 % nahm sie sogar zu. Bemerkenswert ist zudem der hohe Anteil an Einrichtungen mit voller Weiterbildungsermächtigung über 5 Jahre (96,8 %) unter Berücksichtigung eines breiten Spektrums an gewährleisteten Ausbildungsinhalten – von operativen Prozeduren über interdisziplinäre Notfalltherapien bis hin zur psychosomatischen Grundversorgung.

Die Befürchtung, dass im Zuge des KHVVG bzw. Krankenhausreformanpassungsgesetz/KHAG erhebliche Weiterbildungskapazitäten wegfallen könnten, relativiert sich einigermaßen durch die vorliegenden Daten. Die Reform betrifft nach aktuellen Modellierungen vor allem Belegabteilungen, die derzeit nur einen geringen Anteil der Weiterbildungsstellen bereitstellen [3]. Da nahezu alle weiterbildungsaktiven Kliniken Fallzahlen oberhalb der vorgesehenen Mindestmengen aufweisen, ist nicht von einem strukturell bedingten Wegfall wesentlicher Weiterbildungskapazitäten auszugehen.

Zu den Limitationen der Studie gehören die moderate Rücklaufquote sowie die unterschiedliche Beteiligung zwischen universitären (80 %) und nichtuniversitären Einrichtungen (47,4 %). Zudem beruhen alle Angaben auf freiwilligen Selbstauskünften der Einrichtungsleitungen. Zukünftige Untersuchungen könnten durch ergänzende Datenquellen – z. B. Kammerstatistiken, Qualitätsberichte der Krankenhäuser oder bundesweite Register – weiter präzisiert werden.

## Fazit für die Praxis

- Die fachärztliche Weiterbildung in der HNO-Heilkunde wird in Deutschland flächendeckend durch stationäre Einrichtungen gewährleistet.
- Die erhobenen Daten zeigen stabile Strukturen, hohe Behandlungs- und Eingriffszahlen sowie eine weit überwiegende vollständige Weiterbildungsermächtigung.
- Auch unter Berücksichtigung der potenziellen Auswirkungen des Krankenhausversorgungsverbesserungsgesetzes (KHVVG)/Krankenhausreformanpassungsgesetzes (KHAG) ist davon auszugehen, dass die stationären Leistungserbringer weiterhin eine tragende Rolle in der HNO-Facharztweiterbildung einnehmen und somit einen wesentlichen Beitrag zur Sicherstellung der medizinischen Versorgung leisten.

## Data Availability

Die erhobenen Datensätze können auf begründete Anfrage in anonymisierter Form beim korrespondierenden Autor angefordert werden.
